# Association of variant vitamin statuses and tuberculosis development: a systematic review and meta-analysis

**DOI:** 10.1080/07853890.2024.2396566

**Published:** 2024-09-02

**Authors:** Yiqing Zhou, Qian Wu, Fei Wang, Songhua Chen, Yu Zhang, Wei Wang, Chenxi Huang, Kui Liu, Bin Chen

**Affiliations:** a School of Public Health, Hangzhou Medical College, Hangzhou, China; b Department of Tuberculosis Control and Prevention, Zhejiang Provincial Center for Disease Control and Prevention, Hangzhou, China; c School of Public Health, Zhejiang Chinese Medical University, Hangzhou, China; d National Centre for Tuberculosis Control and Prevention, Chinese Center for Disease Control and Prevention, Beijing, China

**Keywords:** Tuberculosis, vitamin, status, deficiency

## Abstract

**Background:**

Several studies have suggested an association between vitamin deficiency and the development of tuberculosis; however, the precise impact remains unclear. This study aimed to elucidate the relationship between distinct vitamin statuses and the occurrence of tuberculosis.

**Materials and methods:**

Retrieval was conducted using several databases without language restrictions to capture the eligible studies on tuberculosis and vitamin status. Pooled odds ratios (ORs), relative risks (RRs), and hazard ratios (HRs) were used with 95% confidence intervals (CIs) to clarify the relationship between the different vitamin statuses (A, B, D, and E) and the occurrence of tuberculosis. Subgroup analysis, sensitivity analysis, meta-regression analysis, and Galbraith plot were performed to determine sources of heterogeneity. Potential publication biases were detected using Begg’s test, Egger’s test, and the trim-and-fill test.

**Results:**

We identified 10,266 original records from our database searches, and 69 eligible studies were considered in this study. The random-effect model showed that people with tuberculosis may exhibit vitamin A deficiency (OR = 10.66, 95%CI: 2.61–43.63, *p* = .001), while limited cohort studies showed that vitamin A supplementation may reduce tuberculosis occurrence. Additionally, vitamin D deficiency was identified as a risk factor for tuberculosis development (RR = 1.69, 95%CI: 1.06–2.67, *p* = .026), and people with tuberculosis generally had lower vitamin D levels (OR = 2.19, 95%CI: 1.76–2.73, *p* < .001) compared to other groups. No publication bias was detected.

**Conclusions:**

This meta-analysis indicated that people with tuberculosis exhibited low levels of vitamins A and D, while vitamin D deficiency was identified as a risk factor for tuberculosis. More randomized controlled interventions at the community levels should be recommended to determine the association between specific vitamin supplementation and tuberculosis onset.

## Introduction

Tuberculosis (TB), caused by *Mycobacterium tuberculosis (MTB)*, is the leading cause of death among infectious diseases [1]. According to the World Health Organization’s report, there were, in 2022, approximately 10.6 million new cases of TB worldwide and 1.3 million deaths attributed to this specific agent [1]. TB often arises from the activation of latent infections while the precise mechanisms remain unclear. Comprehensive intervention primarily focuses on treating latent infections; however, complications such as liver and kidney injuries post-treatment are widespread [2]. Therefore, identifying safe and effective interventions to reduce the risk of TB development has become increasingly imperative.

Previous studies have demonstrated a correlation between vitamins and diseases, particularly infectious diseases [3]. Commonly studied vitamins include A, B, D, and E. Vitamin A regulates immune function and accelerates early sputum smear conversion during TB treatment [4,5]. Vitamin D downregulates inflammatory factors in the cellular immune response, inhibits the excessive bodily inflammatory response of the body, and stimulates the production of natural antimicrobial peptides, thereby reducing the risk of disease onset [6]. Additionally, research suggests that vitamin B supplementation plays a role in the modulation of IFN-γ secretion in response to MTB infection [7]. Vitamin E acts as an antioxidant, scavenging oxygen free radicals, protecting cell membranes, and enhancing lung function in TB cases [8]. Several studies have indicated a general association between nutritional status and TB incidence, with substantial attention directed toward the relationship between micronutrients, such as vitamins, and TB occurrence [9–11]. However, a comprehensive and systematic summary of the relationships between these vitamins and TB remains lacking.

This study aimed to conduct a systematic review and meta-analysis to examine the association between different vitamin statuses (A, B, D, and E) and TB occurrence. Uncovering this association not only informs but also encourages screening for relevant vitamins, facilitating comprehensive TB interventions in the future.

## Materials and methods

Given a systematic review and meta-analysis for this study, this research followed the Preferred Reporting Items for Systematic Reviews and Meta-Analyses (PRISMA) standards [12] (Table S1). This study has been registered in PROSPERO (CRD42024544172).

### Literature search strategy

**Figure 1.**
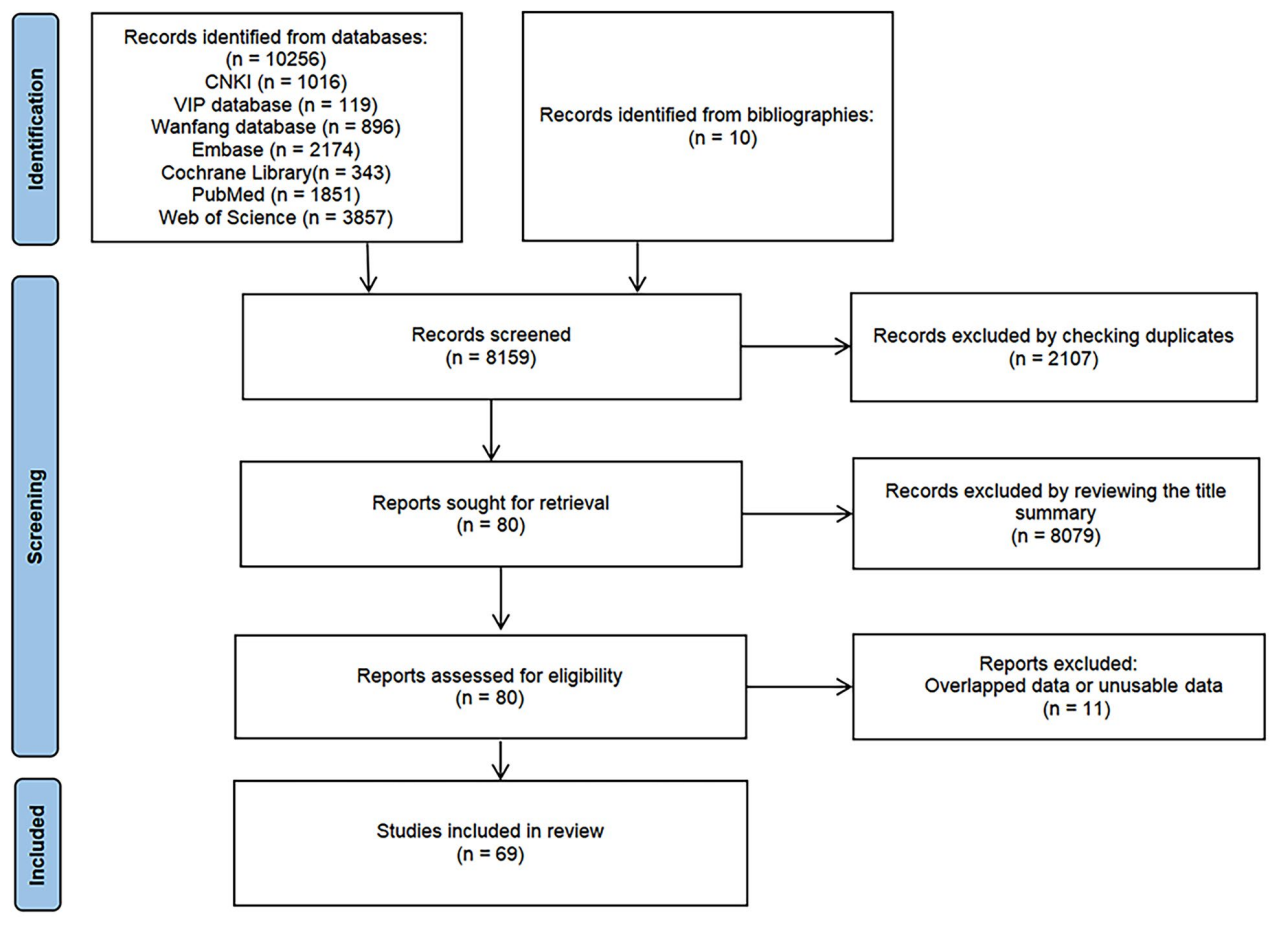
Flow diagram of the study selection.

A computer-based literature search was conducted across multiple databases—PubMed, Web of Science, Embase, Cochrane Library, VIP database, Chinese National Knowledge Infrastructure (CNKI), and Wanfang database—spanning from January 2000 to August 2023 to control the quality and validity of the included literature. The aim was to gather articles reporting the association between various vitamin statuses (A, B, D, and E) and TB. Additionally, relevant references from these articles were also collected. The search terms used included (‘pulmonary tuberculosis’ OR ‘PTB’ OR ‘tuberculosis’ OR ‘TB’) AND (‘vitamin A’ OR ‘vitamin B6’ OR ‘vitamin B12’ OR ‘vitamin D’ OR ‘vitamin E’ OR ‘aquasol A’ OR ‘retinol’ OR ‘cholecalciferol’ OR ‘retinol E’ OR ‘aquasol E’). Detailed search strategies for each database were listed in Table S2. Also, the detailed procedure on literature retrieval was shown in Figure 1.

### Eligibility criteria

The inclusion criteria were as follows: (1) studies reporting the number of individuals supplemented with vitamin A, B, D, or E or detailing their status and TB occurrence without language restrictions; (2) studies presenting two or more original datasets or describing unadjusted and/or adjusted odds ratios (ORs) with corresponding 95% confidence intervals (CIs), unadjusted and/or adjusted relative risks (RRs) with 95%CIs, or unadjusted and/or adjusted hazard ratios (HRs) with 95%CIs; and (3) studies with a design encompassing case-control, cohort, case-cohort, cross-sectional, or randomized controlled trials (RCTs) involving the relationship between vitamin status and TB occurrence.

The exclusion criteria were as follows: (1) meta-analyses, reviews, meeting abstracts, or editorials; (2) studies involving animals; and (3) studies lacking available data, such as those without ORs, RRs, or HRs for secondary or tertiary outcomes.

### Data extraction and quality assessment

Y. Z and C. H determined the eligibility of articles for inclusion. In instances of disagreement Q. W made the final decision. All data were independently extracted from the studies by Y. Z and C. H. The extracted information included details such as the first author, publication year, vitamin type, sample size, characteristics of cases and controls, study year, study design, geographical area, and dose. Quality assessment for case-control and cohort studies was conducted using the Newcastle-Ottawa scale (NOS), which was widely used to assess the quality of case-control and cohort studies [13,14]. Cross-sectional studies were evaluated by adjusting for quality, with a maximum NOS score of 9 [13].

### Statistical analysis

Data processing was performed using Stata version 18. The original studies utilized ORs, RRs, and HRs with 95%CIs to assess the association between deficiencies in vitamins A, B, D, and E and TB among participants. For instance, in cases where articles lacked ORs and 95%CIs but provided the raw number of cases, controls, and exposed individuals in each group, ORs and 95%CIs were calculated during data processing. All data from the included studies were converted into a log (ORs), log (RRs), log (HRs) and their log (95%CIs) values. Statistical heterogeneity was assessed *via Q* and *I*^2^ statistics. In ­heterogeneity tests, when *p* ≤ .1, a random-effects model was used; when *p* > .1, a fixed-effects model was performed [15]. We classified the study designs, such as case-cohort studies, observational studies, and nested case-control studies etc. (excluding RCTs), into cohort studies and case-control studies based on when vitamin levels were measured. We identify heterogeneity through four methods: subgroup analysis which included study start year, sample size, etc; sensitivity analysis which was conducted by sequentially removing individual studies and recalculating the pooled effect; meta-regression analysis which was used to identify sources of heterogeneity; Galbraith plot which was used to detect potential sources of heterogeneity. Publication bias was investigated using Begg’s test, Egger’s linear regression test and the trim-and-fill test [16,17].

## Results

### Study selection and study characteristics

We identified 10,266 potential articles from PubMed, Web of Science, Embase, Cochrane Library, VIP, CNKI, and Wanfang databases. After a careful screening process, 69 articles were included in this systematic review and meta-analysis, spanning the years 2000–2023. Among these, 6 studies reported on vitamin A, 1 on vitamin B12, 1 on vitamin B6, 62 on vitamin D, and 3 on vitamin E. The 69 studies included various study designs, including 3 case-cohort studies [18–20], 36 case-control studies [21–55], 4 nested case-control studies [56–59], 8 cohort ­studies [60–67], 12 cross-sectional studies [68–79], 3 observational studies [80–82], and 3 RCTs [83–85]. The characteristics of these studies are presented in Table 1, with 8 specifically involving individuals with human immunodeficiency virus (HIV). After data collation, 27 articles were conducted in Africa, the region currently experiencing the higher TB prevalence [1]. The NOS scores of the included studies ranged from 5 to 8, indicating moderate quality (Tables S3–S5) and the PEO table was listed (Table S6).

**Table 1.** Characteristics of the studies related to the effects of vitamin and tuberculosis.

Author/ publication year	Vitamin type	Sample size	Characteristic of Cases	Characteristic of Controls	Study year	Study design	Area	Dose
Soh et al. [60]	A	63257	1186 cases who aged 45–74 years	5628 controls who aged 45–74 years	1993–1998	Cohort	Singapore	≥4088 IU/1000 kcal/d
Podel et al. [56]	A	311	30 HIV infected people with TB infection	251 HIV infected people without TB infection	2005–2008	Nested case-control	Haiti	<20 µg/dL
Aibana et al. [59]	A	889	180 pulmonary tuberculosis	709 HHCs of pulmonary tuberculosis	2009–2012	Nested case-control	Peru	<200 µg/L
Tenforde et al. [18]	A	332	77 HIV infected adults with TB infection who aged more than 18 years old	255 HIV infected adults without TB infection who aged more than 18 years old	2005–2007	Case-cohort	Brazil, Haiti, India, Malawi, Peru, South Africa, Thailand, the United States, Zimbabwe	<0.7 μmol/L
Keflie et al. [21]	A	121	62 PTB patients	59 controls	2015	Case-control	Ethiopia	NA
Ramachandran et al. [22]	A	123	47 PTB patients	46 healthy household contacts and 30 healthy ‘normals’	2003	Case-control	India	<30 µg/dL
Tenforde et al. [18]	B6	332	77 HIV infected adults with TB infection who aged more than 18 years old	255 HIV infected adults without TB infection who aged more than 18 years old	2005–2007	Case-cohort	Brazil, Haiti, India, Malawi, Peru, South Africa, Thailand, the United States, Zimbabwe	<19 nmol/L
Tenforde et al. [18]	B12	332	77 HIV infected adults with TB infection who aged more than 18 years old	255 HIV infected adults without TB infection who aged more than 18 years old	2005–2007	Case-cohort	Brazil, Haiti, India, Malawi, Peru, South Africa, Thailand, the United States, Zimbabwe	<148 pmol/L
Tenforde et al. [18]	E	332	77 HIV infected adults with TB infection who aged more than 18 years old	255 HIV infected adults without TB infection who aged more than 18 years old	2005–2007	Case-cohort	Brazil, Haiti, India, Malawi, Peru, South Africa, Thailand, the United States, Zimbabwe	<2.69
Aibana et al. [86]	E	889	180 pulmonary tuberculosis	709 HHCs of pulmonary tuberculosis	2009–2012	Case-control	Lima, Peru	<5 mg/L
Hemilä et al. [85]	E	14573	7286 TB male patients who aged 50–69years old and smoking	7287 controls who aged 50–69years old and smoking	1985–1993	Randomized-control	Finland	50mg/d
Tenforde et al. [18]	D	332	77 HIV infected adults with TB infection who aged more than 18 years old	255 HIV infected adults without TB infection who aged more than 18 years old	2005–2007	Case-cohort	Brazil, Haiti, India, Malawi, Peru, South Africa, Thailand, the United States, Zimbabwe	<20 ng/mL
Venturini et al. [23]	D	858	44 active TB	814 controls	2008–2013	Case-control	Florence, Italy; London, United Kingdom	<10 ng/mL
Ludmir et al. [24]	D	80	39 active TB who aged under 2 years old	41 controls who aged under 2 years old	2010–2012	Case-control	Gaborone, Botswana	<20 ng/mL
Jaimni et al. [25]	D	100	50 adult aged more than 18 years with newly diagnosed sputum positive pulmonary tuberculosis patients	50 age and sex-matched healthy participants as control groups	2014–2016	Case-control	Manipal, India	≤20 ng/ml
Ho-Pham et al. [26]	D	385	166 TB patients	219 controls	2009	Case-control	Ho Chi Minh, Vietnam	<20 ng/mL
Wejse et al. [27]	D	856	362 TB patients	494 controls	2003–2006	Case-control	Guinea-Bissau	≤25 ng/ml
Wilkinson et al. [28]	D	145	103 TB patients	42 controls	NA	Case-control	Asians of Gujarati origin	NA
Gibneyet al. [80]	D	121	40 TB patients	81 controls	2003–2006	Observational	Melbourne, Victoria, Australia	<10 ng/mL
Martinez et al. [61]	D	774	Newborn infants aged 6–10 weeks		2012–2021	Cohort	Cape Town, South Africa	<20 ng/mL
Hammami et al. [29]	D	90	45 extra pulmonary TB	45 controls matched by gender and age	2017–2019	Case-control	Tunisia	<20 ng/mL
Ralph et al. [81]	D	259	167 TB patients	92 controls	NA	Observational	Sabah, Malaysia	NA
Stockdale et al. [30]	D	48	24 highly TB-exposed infected children who aged more than 15 years old	24 highly TB-exposed uninfected children who aged more than 15 years old	2015	Case-control	Gambia	<20 ng/mL
Arnedo-Pena et al. [62]	D	578	33 TB patients	545 residents,staff members and relatives of TB cases	2015–2016	Cohort	Spain	<10 ng/mL
Tessema et al. [31]	D	290	96 PTB patients	194 household contacts	2013–2015	Case-control	Northwest Ethiopia	<20 ng/ml
Zhang et al. [32]	D	239	128 DS-TB and 52 MDR-TB	59 healthy controls	2015–2016	Case-control	Beijing Chest Hospital	<12 ng/mL
Arnedo-Pena et al. [63]	D	572	Contacts of PTB patients		2009–2012	Cohort	Castellon, Spain	NA
Hong et al. [33]	D	376	94 TB patients	282 Korean national survey participants	NA	Case-control	Korea	NA
Maceda et al. [34]	D	72	24 TB patients	48 controls	2013	Case-control	Brazil	<20 ng/mL
Arnedo-Pena et al. [64]	D	198	18 TB patients who aged more than 10 years old	180 controls who aged more than 10 years old	2010–2012	Cohort	Castellon, Spain	<20 ng/ml
Arnedo-Pena et al. [35]	D	93	11 TB patients	82 controls	2009–2010	Case-control	Castellon, Spain	<20 ng/ml
Steenhof et al. [36]	D	38	19 HIV-infected people with TB infection	19 HIV-infected people without TB infection	2008–2010	Case-control	Gaborone, Botswana	<20 ng/mL
Nielsen et al. [37]	D	144	72 TB patients who aged 8–74 years old	72 controls who aged 8–74 years old	2004–2006	Case-control	Greenland	<30 ng/mL
Patterson et al. [65]	D	1509	Latent patients		NA	Cohort	London, UK	<10 ng/mL
Buonsenso et al. [82]	D	57	14 active TB patients who aged more than 14 years old	43 latent TB, non-TB Pneumonia and healthy Children who aged more than 14 years old	2010–2014	Observational	Rome, Italy	<20 ng/mL
Boillat-Blanco et al. [38]	D	425	167 TB patients who aged more than 18 years old	358 sex-and age-matched volunteers who aged more than 18 years old	2013	Case-control	Tanzania	<20 ng/mL
Shukla et al. [39]	D	352	90 newly diagnosed adult MDR-TB cases	180 household controls and 82 non-household controls	NA	Case-control	India	<20 ng/mL
Acen et al. [68]	D	95	56 active TB patients who aged 15–65 years old	39 LTBI and those without TB infection	2019–2020	Cross-sectional	Uganda	<20 ng/mL
Kim et al. [40]	D	362	165 active PTB patients	197 controls	2008–2010	Case-control	Korea	<20 ng/mL
Talat et al. [66]	D	100	Tuberculosis patients and their contacts		2001–2004	Cohort	Karachi,Pakistan	<20 ng/mL
Sita-Lumsden et al. [41]	D	308	178 active TB patients	130 healthy controls	NA	Case-control	London	<8 ng/mL
Williams et al. [42]	D	64	26 active TB patients	38 LTBI	2004–2006	Case-control	London	<8 ng/mL
Sudfeld et al. [83]	D	4000	HIV-infected adults with low vitamin D levels(<30 ng/mL)		2014–2017	Randomized-control	Tanzania	50,000 IU/week (3weeks), 4th week 2000 IU/day
Raheel et al. [53]	D	360	105 TB patients	255 controls	2010–2012	Case-control	Kharian	<25 ng/ml
Balcells et al. [43]	D	262	92 TB patients	139 HHC and 31 non-HHC	2013–2015	Case-control	Santiago, Chile	<20 ng/ml
Sudfeld et al. [84]	D	415	211 HIV-infected people with TB infection	204 HIV-infected people without TB infection	2014–2018	Randomized-control	Tanzania	50,000 IU/week (3weeks), 4th week 2000 IU/day
Dlamini et al. [19]	D	3194	98 cases with end-stage kidney disease (ESKD)	3096 controls with end-stage kidney disease (ESKD)	2010	Case-cohort	Taiwan	NA
Junaid et al. [44]	D	372	260 PTB patients who aged 14–60 years old	112 controls who aged 14–60 years old	2012–2013	Case-control	Lahore, Pakistan	≤8 ng/mL
Nouri-Vaskeh et al. [69]	D	60	30 newly diagnosed TB patients	30 healthy volunteers	2017–2018	Cross-sectional	Tabriz, East Azerbaijan province, North West of Iran	≤20 ng/mL
Workineh et al. [70]	D	253	126 newly diagnosed smear positive TB patients	57 house hold contacts and 70 apparently community controls	2013	Cross-sectional	Northwest Ethiopia	<10 ng/mL
Joo et al. [71]	D	16854	805 PTB patients	16,049 controls	2010–2012	Cross-sectional	Korea	<20 ng/mL
Bater et al. [72]	D	9595	Schoolchildren aged 6–13 years old		2014	Cross-sectional	Mongolia	<10 ng/mL
Friis et al. [73]	D	1569	1223 TB patients	347 controls	2006–2009	Cross-sectional	Mwanza, Tanzania	<20 ng/mL
Manhar et al. [74]	D	296	100 newly diagnosed sputum-positive pulmonary tuberculosis patients of either sex aged 18–60 years	196 age and sex matched hospitalized patients of other medical illnesses	2012–2013	Cross-sectional	North India	≤20 ng/ml
Wang et al. [75]	D	390	299 TB patients	92 controls	2015–2016	Cross-sectional	Qingdao	<20 ng/mL
Aibana et al. [57]	D	889	180 TB patients	709 controls	2009–2012	Nested case-control	Lima, Peru	<20 ng/mL
Ageeru et al. [45]	D	140	70 children between 6 months and 12 years of age who had been recently diagnosed or were already on anti-tuberculous treatment	70 normal children	2019–2020	Case-control	Hyderabad, Telangana, India	<10 ng/mL
Dabla et al. [46]	D	50	25 untreated children with osteoarticular tuberculosis	25 controls	2013–2014	Case-control	NA	<12 ng/mL
Koo et al. [47]	D	202	116 TB patients who aged more than 18 years old	86 controls	2010–2011	Case-control	South Korea	≤10 ng/mL
McArdle et al. [76]	D	166	92 TB patients	74 controls	2011–2014	Cross-sectional	Northwick Park, St Mary’s, Yorkhill, Bristol Royal, Manchester sites	NA
Owolabi et al. [48]	D	181	83 TB patients	46 TST + and 52 TST − HHC	2015	Case-control	Gambia	<10 ng/mL
Yongxue et al. [55]	D	337	208 TB patients	129 controls	2020	Case-control	Qinghai	NA
Mave et al. [58]	D	90	30 HIV infected mothers with TB infection	60 HIV infected mothers without TB infection	2002–2007	Nested case-control	Pune, India	<20 ng/mL
Jubulis et al. [49]	D	178	60 children aged ≤ 5 years with confirmed/probable TB	118 healthy children aged ≤ 5 years	2009–2011	Case-control	Pune, India	≤20 ng/ml
Gupta et al. [20]	D	370	105 HIV infected infants with TB infection who aged 3–4 months	265 HIV infected infants without TB infection who aged 3–4 months	2004–2008	Case-cohort	South Africa, Botswana	<32 ng/mL
Sasidharan et al. [54]	D	51	35 TB patients	16 controls	NA	Case-control	India	NA
Martineau et al. [50]	D	139	84 TB patients	55 controls	2003–2005	Case-control	London, UK	<8 ng/mL
Chaudhary et al. [51]	D	155	30 patients of pulmonary TB and 40 patients with both pulmonary TB and type 2 diabetes	46 patients with type 2 diabetes, 39 non-diabetic healthy controls	NA	Case-control	India	<20 ng/dl
Joshi et al. [52]	D	50	25 TB patients	25 controls	2009–2012	Case-control	Mahavir Hospital, Hyderabad	<10 ng/mL
Nansera et al. [77]	D	100	50 HIV infected infants with TB	50 HIV infected infants without TB	2009	Cross-sectional	south-western Uganda	<12 ng/ml
Martineau et al. [78]	D	370	192 active TB	178 LTBI	2003–2010	Cross-sectional	Cape Town, South Africa	<20 ng/mL
Mastala et al. [79]	D	318	161 TB patients	157 controls	2010	Cross-sectional	Malawi	≤20 ng/mL
Sudfeld et al. [67]	D	1103	ART initiation for 1103 HIV-infected adults		2006–2009	Cohort	Tanzania	<20 ng/mL

### Vitamin A status across groups

**Figure 2.**
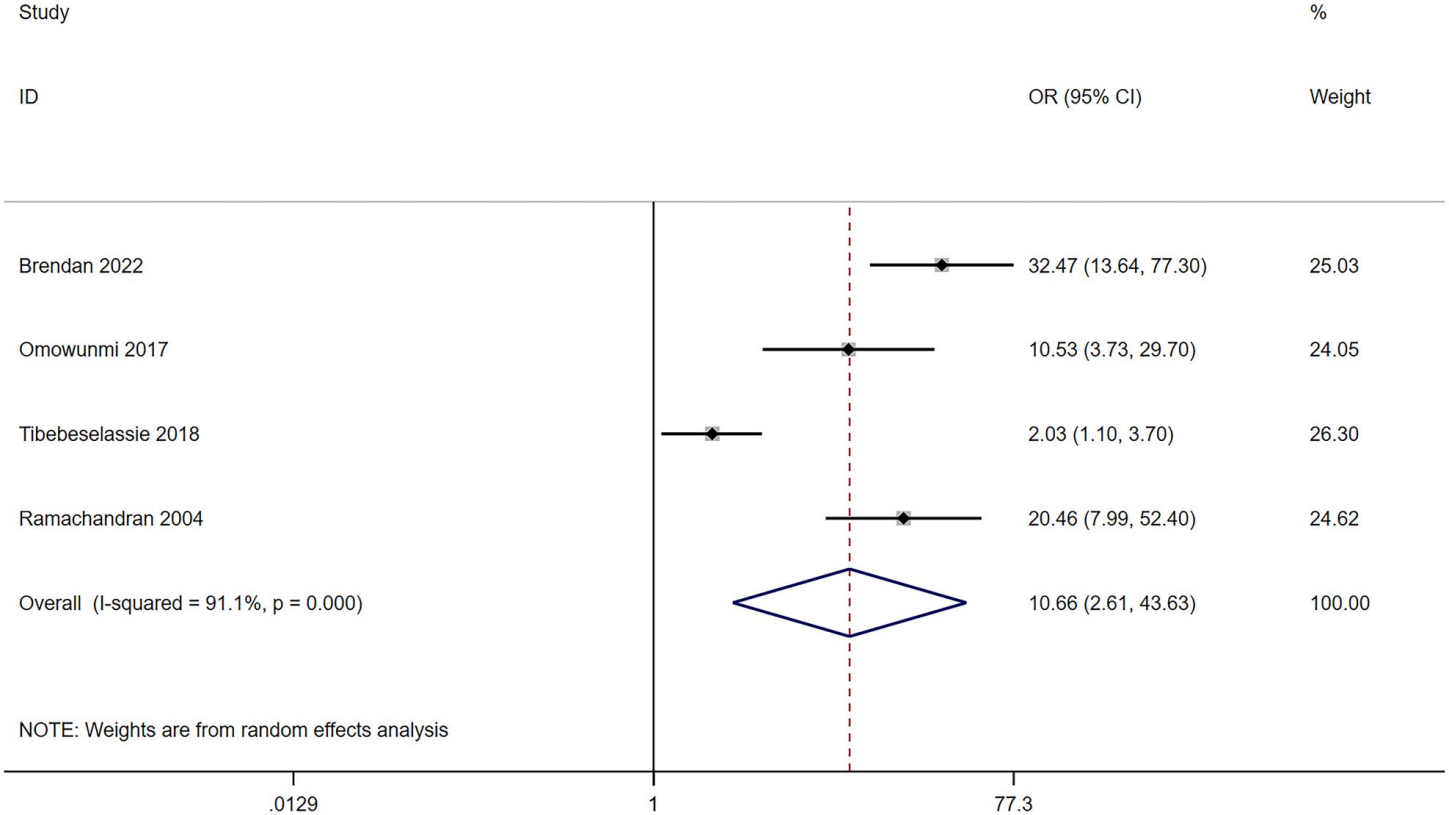
Association between vitamin A and tuberculosis analyzed by the Forest plot. The Forest plots of pooled OR with 95% CI (OR = 10.66, 95% CI: 2.61–43.63; Random-effects model, *p* < .001).

**Figure 3.**
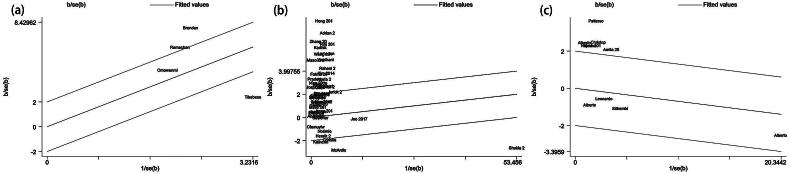
Galbraith plot of association between vitamin and tuberculosis. Each figure represents a unique article in this meta-analysis. The figures outside the three lines were spotted as the outliers and the possible sources of heterogeneity in the analysis pooled from the total available number. (a) Galbraith plot result of vitamin A and tuberculosis; (b) Galbraith plot result of vitamin D and tuberculosis among the case-control studies; (c) Galbraith plot result of vitamin D and tuberculosis among the cohort studies.

Within the subset of 6 studies that explored specific relationships of vitamin A and TB [18,21,22,56,59,60], including 1 cohort study, 1 case-cohort study, 2 nested case-control studies and 2 case-control studies, intriguing findings emerged. The only cohort study, conducted among Chinese individuals aged 45–74 years, indicated that maintaining vitamin A intake above 4088 IU/1000 kcal/day significantly reduced TB risk by 29% (HR = 0.71, 95%CI: 0.59–0.85, *p* < .01) over an average follow-up of 16.9 years. Pooled results from case-control studies indicated a noteworthy association between TB and vitamin A deficiency (OR = 10.66, 95%CI: 2.61–43.63, *p* = .001) (Figure 2), relative to control groups comprising patients with HIV, household contacts, or healthy individuals. Meta-regression analysis, incorporating covariates such as study start year (*p* = .413), HIV status (*p* = .191), sample size (*p* = .413), geographical area (*p* = .137), and NOS (*p* = .258) as covariates. Two studies were identified as outliers in the Galbraith plots (Figure 3a). After omitting these records, the adjusted association of vitamin A and TB showed a lower heterogeneity (*I^2^* = 86.1, *p* = .007) and an increased susceptibility (random-effects model: OR = 3.09, 95%CI: 1.83–5.21, *p* < .001). Then, the sensitivity analysis was carried out in this group, and the pooled effect results showed a good robustness (data not shown).

### Vitamin D status across groups

**Figure 4.**
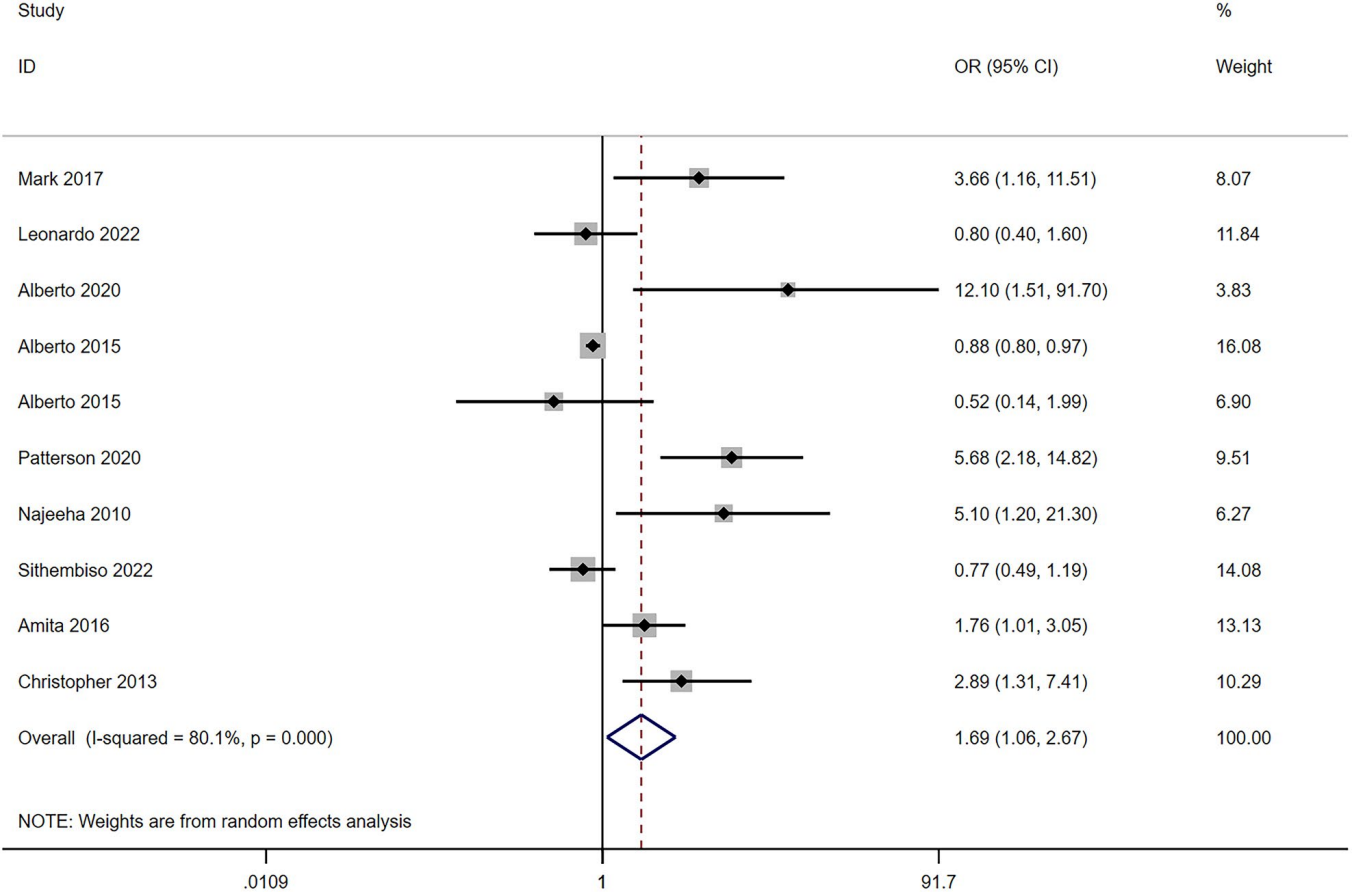
Association between vitamin D and tuberculosis among the cohort studies analyzed by the Forest plot. The Forest plots of pooled RR with 95% CI (RR = 1.69, 95% CI: 1.06–2.67, Random-effects model, *p* = .026).

A total of 62 articles were included to describe the association between vitamin D and TB [18,23–29,33,61,80]. After adjustment, 50 case-control studies, 10 cohort studies, and 2 RCTs were included. Pooled results from case-control studies indicated significantly higher odds of vitamin D deficiency in case groups (OR = 2.19, 95%CI: 1.76–2.73, *I^2^_random-effects model_* = 90.4, *p* < .001) (Figure 4), while also revealing high heterogeneity. Subsequently, we conducted a meta-regression analysis incorporating study year (*p* = .220), HIV status (*p* = .782), sample size (*p* = .101), geographical area (*p* = .008), NOS (*p* = .156), dose (*p* = .061), and timing of vitamin measurements (*p* = .023) as covariates. Further subgroup analysis for case-control studies based on geographical area (*I^2^* = 87.3 Europe; *I^2^* = 85.3 Africa; *I^2^* = 93.3 Asia; *p* < .001) demonstrated that in Africa, Asia, South America and North America, TB cases had a higher prevalence of vitamin D deficiency than controls in different countries (OR = 1.87, 95%CI: 1.21–2.90 for Africa; OR = 2.79, 95%CI: 1.98–3.92 for Asia; OR = 2.07, 95%CI: 1.33–3.21 for South America; OR = 6.50, 95%CI: 1.80–23.49 for North America) (Table 2). However, in a study of European population, no significant association between vitamin D deficiency and TB was observed. For the timing of vitamin measurements, we classified all literature into newly diagnosed cases without initial treatment and other groups including patients with unknown status of anti-TB treatment. Further analysis showed that the newly diagnosed group without treatment had a higher association between vitamin deficiency and TB occurrence (OR = 3.92, 95%CI: 2.20–6.99). Besides, the association in this group was also higher than all studies we included (OR = 2.19, 95%CI: 1.76–2.73) (Table 2). In addition, according to the Galbraith plot for the association between vitamin D and TB in case-control studies, 25 articles were identified as outliers, potentially contributing to heterogeneity (Figure 3b). After excluding these articles, heterogeneity decreased (OR = 1.15, 95%CI: 1.03–1.28, *I^2^_random-effects model_* = 28.6, *p* = .092).

**Table 2.** Subgroup analysis of the association between vitamin D and tuberculosis among case-control studies.

			Odds ratio			Heterogeneity		
Vitamin	Subgroup analysis type	No. of studies	*OR[95%CI]*	*P_OR_*	M	*I*^2^(%)	*P_H_*	*P_E_*
D	All studies	50	2.19[1.76,2.73]	<0.001	R	90.4	<0.001	<0.001
	subgroup analyses by area							
	Europe	7	1.89[0.94,3.79]	0.075	R	87.3	<0.001	0.118
	Africa	20	1.87[1.21,2.90]	0.005	R	85.3	<0.001	0.943
	Asia	17	2.79[1.98,3.92]	<0.001	R	93.3	<0.001	<0.001
	Oceania	1	0.41[0.19,0.90]	0.026	NA	NA	NA	NA
	South America	3	2.07[1.33,3.21]	0.001	F	0.0	0.521	NA
	North America	1	6.50[1.80,23.49]	0.004	NA	NA	NA	NA
	Subgroup analyses by timing of vitamin measurements							
	Newly diagnosed cases without initial treatment	13	3.92[2.20,6.99]	<0.001	R	87.8	<0.001	<0.001
	Others	37	1.82[1.46,2.26]	<0.001	R	88.0	<0.001	<0.001

**Figure 5.**
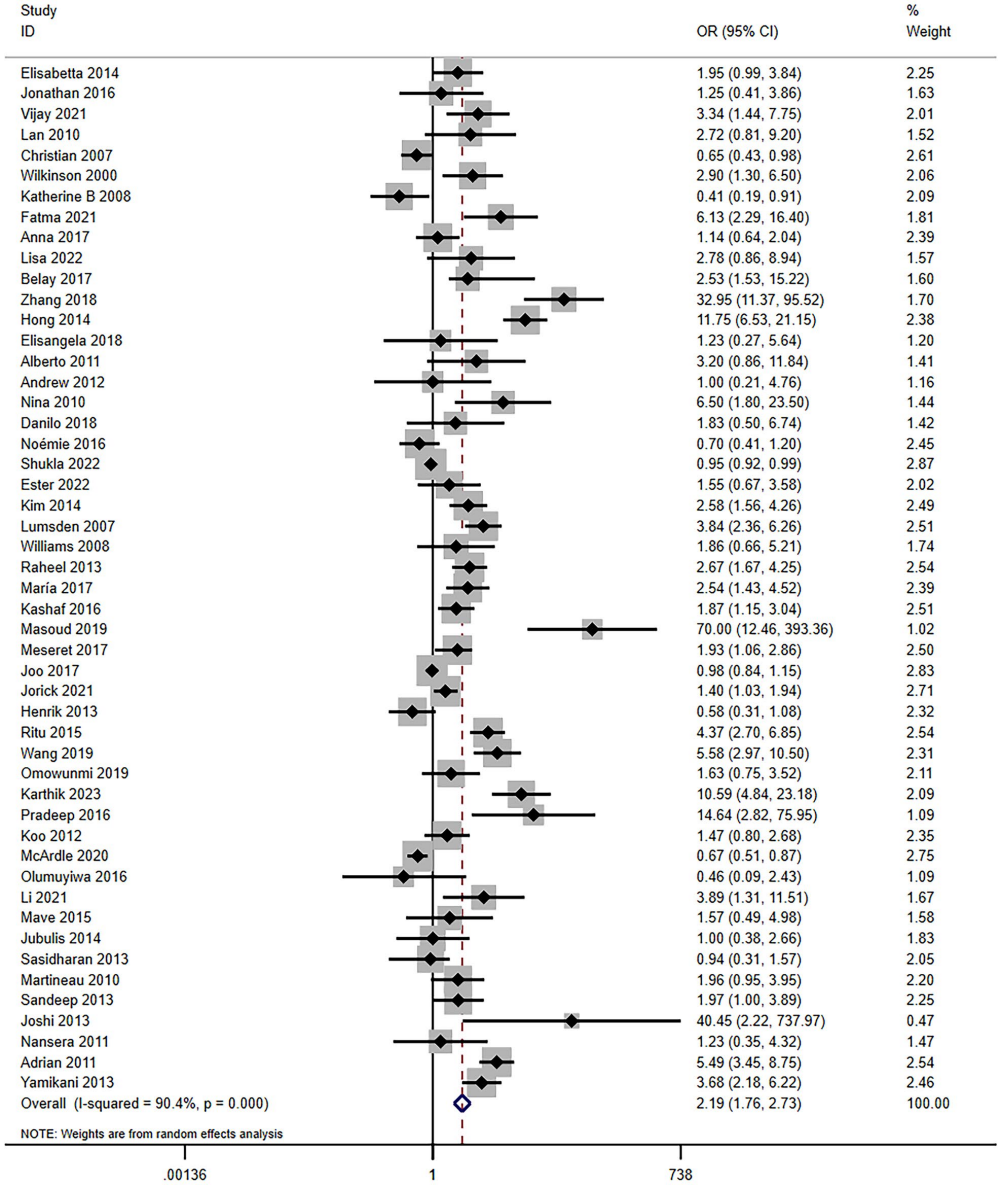
Association between vitamin D and tuberculosis among the case-control studies analyzed by the Forest plot. The Forest plots of pooled OR with 95% CI (OR = 2.19, 95% CI: 1.76–2.73; Random-effects model, *p* < .001).

Among the cohort studies, vitamin D deficiency was identified as a risk factor for TB occurrence (RR = 1.69, 95%CI: 1.06–2.67, *p* = .026) (Figure 5), with high heterogeneity (*I^2^* = 80.1). Subsequently, we conducted a meta-regression analysis incorporating study year (*p* = .212), HIV status (*p* = .245), sample size (*p* = .791), geographical area (*p* = .264), and NOS (*p* = .063) as covariates. According to the Galbraith plot for the association between vitamin D and TB in the cohort studies, 6 articles were identified as outliers (Figure 3c). After adjustment, the association between vitamin D and TB remained significant (fixed-effects model: RR = 0.87, 95%CI: 0.79–0.96, *p* = .004) with lower heterogeneity. Subsequently, a sensitivity analysis was performed for each group. Notably, a RCT observed no significant difference in the total incidence rate of TB between the vitamin D_3_ supplementation group and the placebo group (HR = 0.78, 95%CI: 0.54–1.13, *p* = .19). Sensitivity analysis was conducted for each group (case-control studies and cohort studies), and the pooled effect results showed a good robustness (data not shown).

### Vitamins B and E status across groups

A previous study has examined the effects of vitamins B_12_ and B_6_ on TB [18]. The only study conducted among HIV-infected patients initiating antiretroviral therapy, aged over 18 years, across nine countries indicated vitamin B_12_ intake below 148 pmol/L and vitamin B_6_ intake below 19 nmol/L were not identified as risk factors for TB (HR = 0.82, 95%CI: 0.11–6.11 for vitamin B_12_; HR = 0.75, 95%CI: 0.30–1.89 for vitamin B_6_) which showing that supplementation with vitamins B_12_ and B_6_ does not contribute to reduction in the incidence of TB. Regarding vitamin E, three articles were included in this study [18,85,86]. In a RCT study, vitamin E supplementation (50 mg/d) was found to be ineffective in reducing the incidence of TB among male smokers aged 50–69 years old (HR = 1.18, 95%CI: 0.86–1.59), with vitamin C intake significantly altering the effectiveness of vitamin E. Furthermore, vitamin E supplementation increased the risk of TB by 72% among heavy smokers who concurrently took vitamin C at 90 mg/d. A case-control study evaluated three isomers of vitamin E (α-tocopherol, γ-tocopherol and δ-tocopherol) and their relationship to TB risk. The study was conducted among household contacts of TB cases, revealed that a deficiency in α-tocopherol (<5mg/L) elevated the risk of TB (OR = 1.59; 95%CI: 1.02–2.50; *p* = .04). For δ-tocopherol, household contacts in the lowest tertile were also at an increased risk of progressing to TB disease compared with the highest tertile (OR = 2.29; 95%CI: 1.29–4.09; *p* = .005). The baseline concentration of γ-tocopherol, however, was not correlated with the incidence of TB. Nevertheless, a case-cohort study exploring the relationship between vitamin E and TB among HIV-infected patients from nine countries suggested that vitamin E deficiency may act as a protective factor against TB (RR = 0.38, 95%CI: 0.15–0.97).

### Potential publication bias

**Figure 6.**
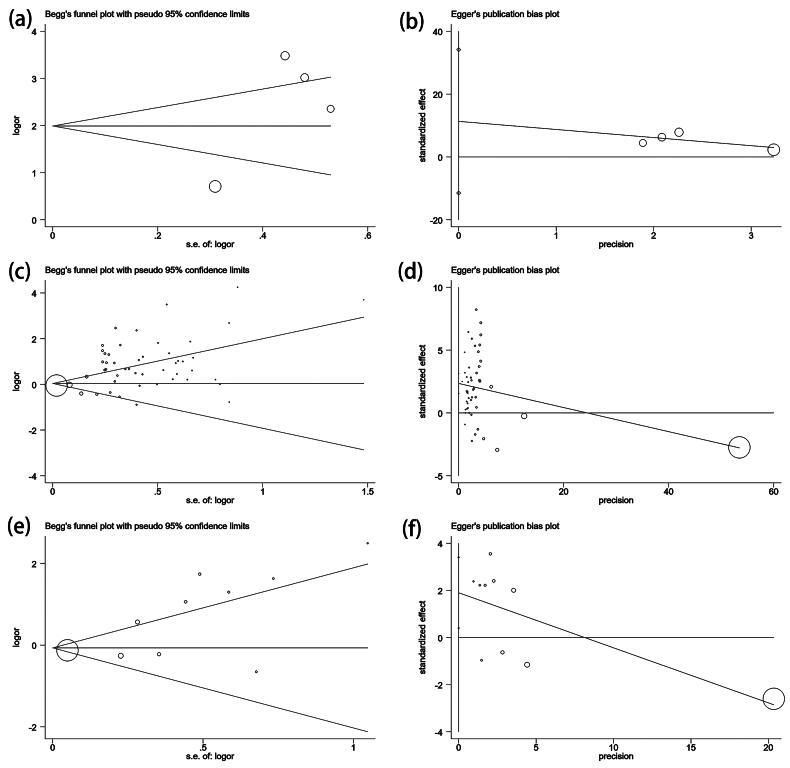
Begg’s funnel plot and egger’s linear regression test of the association between vitamin and tuberculosis. Begg’s funnel plot is used to detect potential publication bias in which a symmetric funnel shape means no publication bias. Egger’s linear regression test is used to quantify the potential presence of publication bias; (a and b) vitamin A: No publication bias has been found from 4 inclusive studies about the association between vitamin A and tuberculosis by Begg’s test and egger’s test, respectively; (c and d) vitamin D (case-control): no publication bias has been found from 50 inclusive studies about the association between vitamin D and tuberculosis by Begg’s test and egger’s test, respectively. (e and f) vitamin D (cohort): publication bias has been found from 10 inclusive studies about the association between vitamin D and tuberculosis among the cohort studies by Begg’s test and egger’s test, respectively.

To assess potential publication bias, we employed Begg’s funnel plots and Egger’s linear regression test (Figure 6(a,b) for vitamin A among case-control studies; Figure 6(c,d) for vitamin D among case-control studies; Figure 6(e,f) for vitamin D among cohort studies). Both Begg’s and Egger’s test yielded consistent results, indicating an absence of publication bias for vitamin A (*P_B_* = 0.806, *P_E_* = 0.290). Similarly, in the case of vitamin D among case-control studies, Begg’s test (*P_B_* = 0.639) revealed no publication bias. To enhance the robustness of these findings, we conducted trim-and-fill tests, which further validated the lack of publication bias in our analyses (data not shown).

## Discussion

Vitamins, particularly A and D, have been shown to play an important role in the adjuvant treatment of TB [3]. Research suggested that vitamin A might enhance the immune system’s ability to combat diseases by reducing cholesterol levels in TB-infected cells, given MTB’s reliance on cholesterol as a nutritional requirement [87]. Additionally, research indicated that maintaining ­adequate vitamin D levels could enhance respiratory function and potentially prevent TB [88]. Moreover, a cross-sectional study conducted in the United States revealed an association between vitamin D deficiency and MTB infection, regardless of HIV status. Furthermore, the occurrence of TB was inversely related to seasonal changes in serum 25-hydroxyvitamin D levels [78]. Despite these compelling insights, systematic reviews, and meta-analyses on the association between vitamin statuses (A, B, D, and E) and TB remained limited. Our study thus assumed a critical role, poised to guide efficient vitamin selection for comprehensive interventions in future TB management.

Vitamin A enhances the resistance of epithelial cells, and its deficiency can lead to reducing respiratory mucosal barrier function and decreasing macrophage and phagocytic activities, thereby weakening the body’s ability to resist infection [89]. In addition, vitamin A supplementation has been found to improve the cellular immune function in TB and increase the rate of lymphocyte transformation. Experimental studies have demonstrated that the bioactive form of vitamin A, all-trans retinoic acid (ATRA), can inhibit the growth of macrophage-toxic MTB [90,91]. The synergistic effects of vitamin D and ATRA on the inhibition of mycobacterial entry and macrophage survival may be achieved by rescuing phagocytic maturation and stasis [91]. All case-control studies showed that vitamin A deficiency was common in TB, suggesting that vitamin A supplementation should be encouraged during treatment [21,22,56,59]. Vitamin A deficiency was often accompanied by malnutrition, which was often closely related to the occurrence of TB. Limited cohort studies have suggested that vitamin A supplementation (≥4,088 IU/1000 kcal/d) can reduce the risk of TB in middle-aged and older individuals by 29% [60]. Therefore, RCTs should be conducted to explore whether vitamin A played a role in the occurrence or activation of infections. However, considering the fat-soluble characteristics of vitamin A, the choice of intake intervention dose and intervention time must be considered, to maximize the protective effect while considering economic benefits.

Considering the inconsistencies among previous studies on vitamin D, we explored its relationship with TB from different design perspectives, including case-control and cohort studies. Results of all case-control studies have shown that vitamin D deficiency is common in patients and maybe a possible risk factor for TB development in comprehensive cohort studies. Additionally, our further results from subgroup analysis revealed a heightened association between vitamin D deficiency and TB development among newly diagnosed patients without treatment. TB, characterized as a wasting disease, frequently occurs alongside severe vitamin deficiency. Thus, vitamin D level typically presented lower before the initiation of anti-TB therapy [66,70,92]. We speculated that individuals undergoing anti-TB treatment may receive more nutritional supplementation, potentially reducing this specific association. Besides, increasing evidence showed vitamin D could accelerate the transformation of sputum smear-positive TB to negative TB, inhibit the release of proinflammatory factors under antigen stimulation, improve lung function, and enhance immunity. In addition, vitamin D could promote the release of a molecule called IFN-gamma by adaptive immune cells (called T cells) and activate innate immune cells (macrophages) to attack invading MTB [93–96]. Therefore, adding vitamin D supplementation to subsequent anti-TB treatment holds significance. In our study, three studies conducted in HIV-infected populations showed that vitamin D deficiency was a risk factor for TB, whereas no such effect was observed in other populations. Also, limited RCTs indicated that vitamin D supplementation in HIV populations did not affect the overall incidence of TB in HIV-infected patients with low vitamin D levels who began ART [84]. Given the conflicting results from available findings, we should determinate and verify the rationality of vitamin D supplementation in people with TB and HIV infection in the future. Due to the limited number of RCTs, the next phase could involve exploring potential mechanisms and causes through multicenter population studies at the molecular level.

Limited research has explored the relationship between vitamins B and E and TB. Vitamin B includes vitamins B_1_, B_6_, and B_12_. Vitamin B_1_ plays an important role in macrophages and affects neutrophil movement. Vitamin B_6_ can release the cytokines or chemokines responsible for NK cell reactivity. In addition, vitamin B_12_ increases lymphocyte counts, improves abnormal CD4/CD8 ratios, and enhances NK cell activity [7]. A single case-cohort study found no evidence that deficiencies in vitamins B_6_ and B_12_ were risk factors for TB in HIV-infected individuals [18]. Further RCTs or cohort studies are needed to explore their relationship and the potential impact of vitamin B supplementation on TB. Vitamin E deficiency may increase the risk of TB in household contacts with TB [86]. During the immune response to MTB invasion, neutrophils are stimulated to produce excess reactive oxygen species, leading to cellular damage and chronic inflammation. Vitamin E reduces oxidative damage in lung tissues by scavenging free radical [8]. A case-control study demonstrated TB patients’ deficiency in vitamin E [86], possibly linked to its depletion in diseases with poor fat absorption, such as TB. In addition, patients with TB may experience vitamin E deficiency due to malnutrition. Low levels of α-tocopherol and δ-tocopherol isomers are associated with an increased risk of TB among TB household contacts. These findings suggested that the assessment of vitamin E concentration in high-risk groups or vitamin E supplementation during follow-up treatment may play a role in TB control. In case-cohort studies, the absence of γ-isomers may act as a protective factor for TB in HIV-infected individuals, contradicting the results of case-control studies. This discrepancy might be due to differences in study populations, with the case-control study focusing on household contacts and the case-cohort study on HIV-infected individuals. Further exploration of the roles of various vitamin E isomers in TB incidence across different populations is warranted. However, results from an RCT involving a 50-69 year old smoking population indicated that vitamin E supplementation had no significant effect on TB incidence, suggesting that smokers might have been deficient in vitamin E [85]. Epidemiological investigations revealed that low antioxidant intake, such as vitamin E, increased the risk of atherosclerosis and other diseases. However, a meta-analysis suggested that vitamin E supplementation exceeding 150 mg/d may elevate the mortality rate [97]. Analysis of the Alpha-Tocopherol, Beta-Carotene Cancer Prevention (ATBC) cohort study indicated that low vitamin E supplementation (<50 mg/d) might have detrimental effects on specific populations. Therefore, caution should be exercised regarding the use of vitamin E supplementation for TB prevention and treatment [85].

Our meta-analysis has certain limitations. First, the definitions of various vitamin deficiency such as vitamin D was controversial before 2019 which may cause some influence of our findings. Second, due to the limited existing research studying vitamin A, B, and E, there was insufficient data, making it impossible for us to draw precise conclusions. Nevertheless, our meta-analysis has several merits. First, it addressed a critical gap in current research, contributing valuable insights to the field. Second, in contrast to previous meta-analyses comparing the blood vitamin D status of TB and control groups, our study incorporated more recent literature, which enhanced the statistical robustness of our findings [98]. Third, we used all available research data to provide a comprehensive exploration of the relationship between vitamins and the occurence of TB.

## Conclusions

This meta-analysis indicated that people with tuberculosis exhibited low levels of vitamins A and D, while vitamin D deficiency was identified as a risk factor for tuberculosis. Given the limited research, randomized controlled interventions at the community level are recommended to determine the association between specific vitamin supplementation and tuberculosis onset.

## Supplementary Material

Supplemental Material

## Data Availability

The data will be made available on request from the corresponding author.
