# Supplementary material for: Association of variant vitamin statuses and tuberculosis development: a systematic review and meta-analysis
Source: Ann Med. 2024 Sep 2;56(1):2396566. doi: 10.1080/07853890.2024.2396566 (PMC11370680; doi:10.1080/07853890.2024.2396566)
Supplement: Supplemental Material [file IANN_A_2396566_SM1118.zip › suppl_data/Table S2.docx]

**Table S2 The search strategy of the seven databases (PubMed, Web of science, Embase, Cochrane Library, VIP, Chinese National Knowledge Infrastructure, and Wanfang databases)**

| Database | Searches |
| --- | --- |
| PubMed | ((((((((((vitamin A[MeSH Terms]) OR (vitamin B6[MeSH Terms])) OR (vitamin B12[MeSH Terms])) OR (vitamin D[MeSH Terms])) OR (vitamin E[MeSH Terms])) OR (aquasol A[MeSH Terms])) OR (retinol[MeSH Terms])) OR (cholecalciferol[MeSH Terms])) OR (aquasol E[MeSH Terms])) AND ((tuberculosis[MeSH Terms]) OR (pulmonary tuberculosis[MeSH Terms])) |
| Web of science | (ALL=(vitamin A OR vitamin B6 OR vitamin B12 OR vitamin D OR vitamin E OR aquasol A OR retinol OR cholecalciferol OR retinol E OR aquasol E)) AND (ALL=(pulmonary tuberculosis OR PTB OR tuberculosis OR TB)) |
| Embase | ('vitamin a':ti,ab,kw OR 'vitamin b6':ti,ab,kw OR 'vitamin b12':ti,ab,kw OR 'vitamin d':ti,ab,kw OR 'vitamin e':ti,ab,kw OR 'aquasol a':ti,ab,kw OR retinol:ti,ab,kw OR cholecalciferol:ti,ab,kw OR 'retinol e':ti,ab,kw OR 'aquasol e':ti,ab,kw) AND ('pulmonary tuberculosis':ti,ab,kw OR ptb:ti,ab,kw OR tuberculosis:ti,ab,kw OR tb:ti,ab,kw) |
| Cochrane Library | vitamin A OR vitamin B6 OR vitamin B12 OR vitamin D OR vitamin E OR aquasol A OR retinol OR cholecalciferol OR retinol E OR aquasol E in Title Abstract Keyword AND pulmonary tuberculosis OR PTB OR tuberculosis OR TB in Title Abstract Keyword |
| VIP database | (K=pulmonary tuberculosis OR PTB OR tuberculosis OR TB ) AND (K= vitamin A OR vitamin B6 OR vitamin B12 OR vitamin D OR vitamin E OR aquasol A OR retinol OR cholecalciferol OR retinol E OR aquasol E) |
| CNKI database | (SU %= 'tuberculosis' OR SU %= 'pulmonary tuberculosis' OR SU %= 'PTB' OR SU %= 'TB') AND (SU %= 'vitaminA' OR SU %= 'vitaminB6' OR SU %= 'vitaminB12'' OR SU %= 'vitaminD' OR SU %= 'vitaminE' OR SU %= 'aquasol A' OR SU %= 'retinol' OR SU %= 'cholecalciferol' OR SU %= 'retinol E' OR SU %= 'aquasol E') |
| Wanfang database | (vitamin A OR vitamin B6 OR vitamin B12 OR vitamin D OR vitamin E OR aquasol A OR retinol OR cholecalciferol OR retinol E OR aquasol E) AND (pulmonary tuberculosis OR PTB OR tuberculosis OR TB) |
