# Supplementary material for: Association of variant vitamin statuses and tuberculosis development: a systematic review and meta-analysis
Source: Ann Med. 2024 Sep 2;56(1):2396566. doi: 10.1080/07853890.2024.2396566 (PMC11370680; doi:10.1080/07853890.2024.2396566)
Supplement: Supplemental Material [file IANN_A_2396566_SM1118.zip › suppl_data/Table S3.docx]

**Table S3 Newcast****le-Ottawa quality assessment scores of the included case-control studies**

| Study title | Is the case  definition  adequate | Representativeness  of the cases | Selection  of Controls | Definition  of  Controls | Comparability of  cases and controls on  the basis of the  design or analysis | Ascertainment  of exposure | Same method of  ascertainment  for cases and  controls | Non-  Response  rate | NOS  score |
| --- | --- | --- | --- | --- | --- | --- | --- | --- | --- |
| Brendan et al./2022 | 1 | 1 | 0 | 1 | 2 | 1 | 1 | 0 | 7 |
| Omowunmi et al./2017 | 1 | 1 | 1 | 0 | 2 | 1 | 1 | 1 | 8 |
| Tibebeselassie et al./2018 | 1 | 1 | 1 | 0 | 2 | 1 | 1 | 0 | 7 |
| Ramachandran et al./2004 | 1 | 0 | 0 | 0 | 2 | 1 | 1 | 0 | 5 |
| Omowunmi et al./2018 | 1 | 1 | 1 | 1 | 1 | 1 | 1 | 0 | 7 |
| Elisabetta et al./2014 | 1 | 1 | 1 | 0 | 2 | 1 | 1 | 0 | 7 |
| Jonathan et al./2016 | 1 | 1 | 1 | 0 | 2 | 1 | 1 | 0 | 7 |
| Vijay et al./2021 | 1 | 0 | 0 | 0 | 2 | 1 | 1 | 0 | 5 |
| Lan et al./2010 | 1 | 1 | 1 | 0 | 2 | 1 | 1 | 0 | 7 |
| Christian et al./2007 | 1 | 1 | 1 | 0 | 2 | 1 | 1 | 0 | 7 |
| Wilkinson et al./2000 | 1 | 1 | 0 | 1 | 2 | 1 | 1 | 0 | 7 |
| Katherine et al./2008 | 1 | 1 | 0 | 1 | 1 | 1 | 1 | 0 | 6 |
| Fatma et al./2021 | 1 | 1 | 1 | 1 | 2 | 0 | 1 | 0 | 7 |
| Anna et al./2017 | 1 | 1 | 0 | 1 | 2 | 0 | 1 | 0 | 6 |
| Lisa et al./2022 | 1 | 1 | 1 | 1 | 2 | 1 | 1 | 0 | 8 |
| Belay et al./2017 | 1 | 1 | 1 | 1 | 2 | 1 | 1 | 0 | 8 |
| Zhang et al./2018 | 1 | 1 | 1 | 1 | 1 | 0 | 1 | 0 | 6 |
| Hong et al./2014 | 1 | 1 | 0 | 1 | 1 | 1 | 1 | 0 | 6 |
| Elisangela et al./2018 | 1 | 1 | 1 | 1 | 1 | 1 | 1 | 0 | 7 |
| Arnedo et al./2011 | 1 | 1 | 1 | 0 | 2 | 1 | 1 | 0 | 7 |
| Andrew et al./2012 | 1 | 1 | 0 | 1 | 0 | 1 | 1 | 0 | 5 |
| Nina et al./2010 | 1 | 1 | 1 | 1 | 1 | 1 | 1 | 0 | 7 |
| Danilo et al./2018 | 1 | 1 | 0 | 1 | 2 | 0 | 1 | 0 | 6 |
| Noémie et al./2016 | 1 | 1 | 1 | 1 | 1 | 1 | 1 | 0 | 7 |
| Shukla et al./2022 | 1 | 1 | 1 | 1 | 1 | 0 | 1 | 0 | 6 |
| Kim et al./2014 | 1 | 1 | 0 | 1 | 2 | 0 | 1 | 0 | 6 |
| Lumsden et al./2007 | 1 | 1 | 1 | 1 | 1 | 1 | 1 | 0 | 7 |
| Williams et al./2008 | 1 | 1 | 0 | 1 | 0 | 1 | 1 | 0 | 5 |
| Raheel et al./2013 | 1 | 1 | 1 | 1 | 0 | 1 | 1 | 0 | 6 |
| María et al./2017 | 1 | 1 | 1 | 1 | 0 | 1 | 1 | 0 | 6 |
| Kashaf et al./2016 | 1 | 1 | 0 | 1 | 2 | 0 | 1 | 0 | 6 |
| Omowunmi et al./2019 | 1 | 1 | 1 | 1 | 1 | 1 | 1 | 0 | 7 |
| Karthik et al./2023 | 1 | 1 | 0 | 1 | 2 | 0 | 1 | 0 | 6 |
| Pradeep et al./2016 | 1 | 1 | 0 | 1 | 2 | 0 | 1 | 0 | 6 |
| Hyeon et al./2012 | 1 | 1 | 1 | 1 | 1 | 0 | 1 | 0 | 6 |
| Olumuyiwa et al./2016 | 1 | 1 | 0 | 1 | 1 | 0 | 1 | 0 | 5 |
| Li et al./2016 | 1 | 1 | 0 | 1 | 2 | 0 | 1 | 0 | 6 |
| Mave et al./2015 | 1 | 1 | 1 | 1 | 1 | 0 | 1 | 0 | 6 |
| Jubulis et al./2014 | 1 | 1 | 1 | 1 | 1 | 1 | 1 | 0 | 7 |
| Sasidharan et al./2013 | 1 | 1 | 1 | 1 | 1 | 0 | 1 | 0 | 6 |
| Martineau et al./2010 | 1 | 1 | 1 | 1 | 1 | 1 | 1 | 0 | 7 |
| Sandeep et al./2013 | 1 | 1 | 1 | 0 | 1 | 0 | 1 | 0 | 5 |
| Joshi et al./2013 | 1 | 1 | 0 | 1 | 2 | 0 | 1 | 0 | 6 |
