# Supplementary material for: Association of variant vitamin statuses and tuberculosis development: a systematic review and meta-analysis
Source: Ann Med. 2024 Sep 2;56(1):2396566. doi: 10.1080/07853890.2024.2396566 (PMC11370680; doi:10.1080/07853890.2024.2396566)
Supplement: Supplemental Material [file IANN_A_2396566_SM1118.zip › suppl_data/Table S4.docx]

**Table S4 Modified Newcastle-Ottawa assessment scores for the included cross-sectional studies**

| Study title | Representativeness  of the sample | Sample  size | Ascertainment of  exposure | Non-  respondents | Comparability | Assessment  of outcome | Statistical  test | NOS  score |
| --- | --- | --- | --- | --- | --- | --- | --- | --- |
| Ester et al./2022 | 0 | 1 | 1 | 0 | 1 | 1 | 1 | 5 |
| Masoud et al./2019 | 0 | 1 | 1 | 0 | 2 | 1 | 1 | 6 |
| Meseret et al./2017 | 1 | 1 | 1 | 1 | 1 | 1 | 1 | 7 |
| Mi et al./2017 | 1 | 1 | 1 | 1 | 1 | 1 | 1 | 7 |
| Jorick et al./2021 | 0 | 1 | 1 | 0 | 2 | 1 | 1 | 6 |
| Henrik et al./2013 | 1 | 1 | 1 | 1 | 1 | 1 | 1 | 6 |
| Ritu et al./2015 | 1 | 1 | 1 | 1 | 1 | 1 | 1 | 6 |
| Wang et al./2019 | 1 | 1 | 1 | 1 | 1 | 1 | 1 | 6 |
| McArdle et al./2020 | 0 | 1 | 1 | 0 | 1 | 1 | 1 | 5 |
| Nansera et al./2011 | 1 | 1 | 1 | 0 | 1 | 1 | 1 | 6 |
| Adrian et al./2011 | 0 | 1 | 1 | 0 | 2 | 1 | 1 | 6 |
| Yamikani et al./2013 | 1 | 1 | 1 | 1 | 1 | 1 | 1 | 7 |
