# Supplementary material for: Association of variant vitamin statuses and tuberculosis development: a systematic review and meta-analysis
Source: Ann Med. 2024 Sep 2;56(1):2396566. doi: 10.1080/07853890.2024.2396566 (PMC11370680; doi:10.1080/07853890.2024.2396566)
Supplement: Supplemental Material [file IANN_A_2396566_SM1118.zip › suppl_data/Table S5.docx]

**Table S5 Newcastle-Ottawa quality assessment scores of the included cohort studies**

| Study title | Representative  ness of the  exposed cohort | Selection of the  non-exposed  cohort | Ascertainment  of exposure | Demonstration that  outcome of interest  was not present at  start of study | Comparability | Assessment  of outcome | Was follow-up long  enough for  outcomes to occur | Adequacy  of follow up  of cohorts | NOS  score |
| --- | --- | --- | --- | --- | --- | --- | --- | --- | --- |
| Avril et al./2017 | 1 | 1 | 1 | 0 | 2 | 1 | 1 | 1 | 8 |
| Mark et al./2017 | 1 | 1 | 1 | 0 | 2 | 0 | 0 | 0 | 5 |
| Leonardo et al./2022 | 1 | 1 | 1 | 0 | 2 | 1 | 1 | 0 | 7 |
| Arnedo et al./2020 | 1 | 1 | 1 | 0 | 2 | 0 | 0 | 0 | 5 |
| Arnedo et al./2015 | 1 | 1 | 1 | 0 | 2 | 1 | 1 | 1 | 8 |
| Arnedo et al./2015 | 1 | 1 | 1 | 0 | 2 | 1 | 0 | 0 | 6 |
| Patterson et al./2020 | 1 | 1 | 1 | 0 | 2 | 1 | 1 | 0 | 7 |
| Najeeha et al./2010 | 1 | 1 | 1 | 0 | 2 | 0 | 0 | 0 | 5 |
| Sithembiso et al./2022 | 1 | 1 | 1 | 0 | 2 | 1 | 1 | 1 | 8 |
| Amita et al./2016 | 1 | 1 | 1 | 0 | 2 | 1 | 1 | 0 | 7 |
| Christopher et al./2013 | 1 | 1 | 1 | 0 | 2 | 1 | 1 | 0 | 7 |
