# Supplementary material for: Association of variant vitamin statuses and tuberculosis development: a systematic review and meta-analysis
Source: Ann Med. 2024 Sep 2;56(1):2396566. doi: 10.1080/07853890.2024.2396566 (PMC11370680; doi:10.1080/07853890.2024.2396566)
Supplement: Supplemental Material [file IANN_A_2396566_SM1118.zip › suppl_data/Table S6.docx]

**Table S6 PEO table**

| Number | Study title | Population | Exposure | Outcome |
| --- | --- | --- | --- | --- |
| 1 | Avril et al./2017 | People who aged 45-74 years | vitamin A intake ≥ 4088IU/1000kcal/day | Occurrence of tuberculosis |
| 2 | Mark et al./2017 | HIV infected adults who aged more than 18 years old | vitamin A intake < 0.7μmol/L | Occurrence of tuberculosis |
| 2 | Mark et al./2017 | HIV infected adults who aged more than 18 years old | vitamin D intake < 20 ng/mL | Occurrence of tuberculosis |
| 2 | Mark et al./2017 | HIV infected adults who aged more than 18 years old | vitamin E intake < 2.69 | Occurrence of tuberculosis |
| 2 | Mark et al./2017 | HIV infected adults who aged more than 18 years old | vitamin B_6_ intake < 19 nmol/L | Occurrence of tuberculosis |
| 2 | Mark et al./2017 | HIV infected adults who aged more than 18 years old | vitamin B_12_ intake < 148 pmol/L | Occurrence of tuberculosis |
| 2 | Mark et al./2017 | HIV infected adults who aged more than 18 years old | vitamin Se intake < 85 µg/L | Occurrence of tuberculosis |
| 3 | Leonardo et al./2022 | Newborn infants aged 6-10 weeks | vitamin D intake < 20 ng/mL | Occurrence of tuberculosis |
| 4 | Arnedo et al./2020 | LTBI - negative participants | vitamin D intake < 10 ng/mL | Occurrence of tuberculosis |
| 5 | Arnedo et al./2015 | Contacts of PTB patients | NA | Occurrence of tuberculosis |
| 6 | Arnedo et al./2015 | People who aged more than 10 years old | vitamin D intake < 20 ng/ml | Occurrence of tuberculosis |
| 7 | Patterson et al./2020 | LTBI | vitamin D intake < 10 ng/mL | Occurrence of tuberculosis |
| 8 | Najeeha et al./2010 | Tuberculosis patients and their contacts | vitamin D intake < 20 ng/mL | Occurrence of tuberculosis |
| 9 | Sithembiso et al./2022 | People who with end-stage kidney disease (ESKD) | NA | Occurrence of tuberculosis |
| 10 | Amita et al./2016 | HIV infected infants who aged 3-4 months | vitamin D intake < 32 ng/mL | Occurrence of tuberculosis |
| 11 | Christopher et al./2013 | HIV-infected adults who with ART initiation | vitamin D intake < <20 ng/mL | Occurrence of tuberculosis |
